# Estradiol and Estrogen-like Alternative Therapies in Use: The Importance of the Selective and Non-Classical Actions

**DOI:** 10.3390/biomedicines10040861

**Published:** 2022-04-06

**Authors:** Szidónia Farkas, Adrienn Szabó, Anita Emőke Hegyi, Bibiána Török, Csilla Lea Fazekas, Dávid Ernszt, Tamás Kovács, Dóra Zelena

**Affiliations:** 1 Centre for Neuroscience, Szentágothai Research Center, Institute of Physiology, Medical School, University of Pécs, 7624 Pécs, Hungary; farkasaszidonia@gmail.com (S.F.); szabo.adrienne93@gmail.com (A.S.); anita.e.hegyi@gmail.com (A.E.H.); torok.bibiana@gmail.com (B.T.); ghalla195@gmail.com (C.L.F.); ernszt.david@pte.hu (D.E.); tamas.kovacs@aok.pte.hu (T.K.); 2 János Szentágothai School of Neurosciences, Semmelweis University, 1085 Budapest, Hungary; 3 Department of Dentistry, Oral and Maxillofacial Surgery, Medical School, University of Pécs, 7624 Pécs, Hungary

**Keywords:** estrogen, non-classical actions, tissue-selectivity, alternative therapies, menopause, SERMs, ANGELS, phytoestrogens, osteoporosis

## Abstract

Estrogen is one of the most important female sex hormones, and is indispensable for reproduction. However, its role is much wider. Among others, due to its neuroprotective effects, estrogen protects the brain against dementia and complications of traumatic injury. Previously, it was used mainly as a therapeutic option for influencing the menstrual cycle and treating menopausal symptoms. Unfortunately, hormone replacement therapy might be associated with detrimental side effects, such as increased risk of stroke and breast cancer, raising concerns about its safety. Thus, tissue-selective and non-classical estrogen analogues have become the focus of interest. Here, we review the current knowledge about estrogen effects in a broader sense, and the possibility of using selective estrogen-receptor modulators (SERMs), selective estrogen-receptor downregulators (SERDs), phytoestrogens, and activators of non-genomic estrogen-like signaling (ANGELS) molecules as treatment.

## 1. Introduction

**Figure 1:**
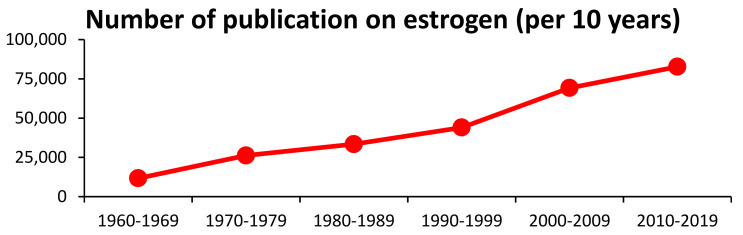
Number of published papers in a 10-year period based on a PubMed search on keyword “estrogen” and dates.

Female sexual hormones, including estrogens, were discovered and characterized in the 1920s and 1930s [1,2,3]. Although 100 years have passed since the first discovery, the interest in estrogen, reflected by the high number of related publications (Figure 1), is still increasing [4].

Even though estrogen hormones affect the function of almost all genes in the mammalian body, they are best known for their major role in reproduction. Estrogens modulate the development of secondary female characteristics [5], and mediate reproduction, the menstrual cycle, sexual behavior, and the emotional background [6,7]. During menopause or after surgical removal of the ovaries (ovariectomy, OVX; a model in animals, but occurs also in humans), the concentration of estrogen decreases, resulting in unpleasant symptoms such as hot flushes, emotional disturbances, memory loss, and more severe problems including osteoporosis [8,9]. Indeed, estrogen helps to develop long bones and pubic epiphyses during puberty. Moreover, it protects the bone structure by inhibiting the degrading osteoclast activity. With these mechanisms, it exerts positive effects on osteoporosis in both estrogen-deficient and post-menopausal-aged women [10,11,12]. The treatment of menopausal symptoms was one of the very first therapeutic applications of estrogens. Nevertheless, estrogen may affect many other physiological processes and plays an important role in their development, and thus, also in the treatment of various diseases [13,14,15].

This review aims to discuss the effects of estrogen in a broader sense, to investigate the importance of non-classical estrogen pathways in the whole body, and to analyze the existing and possible treatment options for improving hormonal replacement therapies (HRT).

## 2. Estrogen Synthesis and Receptors

### 2.1. Forms and Synthesis of Estradiol

**Figure 2:**
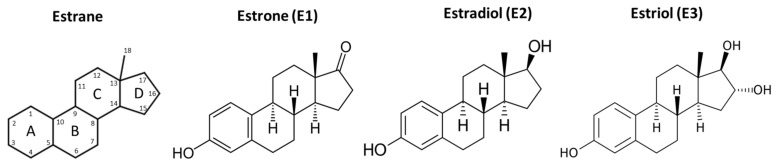
Structure of estrane backbone and the three most important estrogen molecules in the human body.

Estrogen (next to progesterone) is the most important female sex steroid in vertebrates [16]. For its synthesis, the cytochrome P450 aromatase (CYP19) enzyme is essential. This enzyme, catalyzing the rate-limiting step in estrogen biosynthesis, was already detected in invertebrates; however, it shows only 40% amino acid identity with human CYP19 [16]. In humans, there are three important estrogen molecules: estrone (E1), 17β-estradiol (E2), and estriol (E3) [17]. All three compounds are based on an 18 C-atom hydrocarbon backbone called estrane (Figure 2).

Their main structural features are the phenolic ring A, the cyclohexane ring B, ring C with a methyl group in the C13 substitution, and ring D, to which a hydroxyl group (Estradiol) or oxygen (Estrone) is attached at the C17 position.

**Figure 3:**
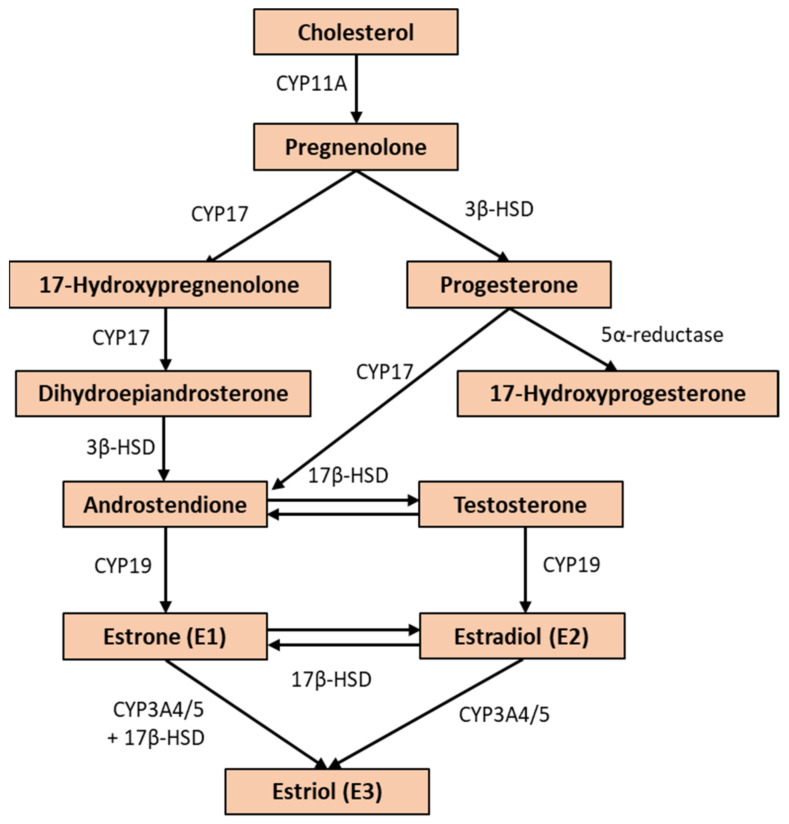
The biosynthesis of estrogen molecules (E1, E2, and E3). The enzymes that catalyze the reactions are: CYP17—17α-hydroxylase; 3β-HSD—3β-hydroxysteroid dehydrogenase; 17β-HSD—17β-hydroxysteroid dehydrogenase; CYP19—aromatase; CYP3A4/5—16α-hydroxylase.

E2 is the most potent estrogen hormone produced in the ovary. Its synthesis in the internal theca of the ovary starts from acetyl-CoA and cholesterol, from which androstenedione, an androgen, is formed through several steps (Figure 3). The androstenedione crosses the basal membrane of the granulosa cells where CYP19 catalyzes E2 synthesis. Furthermore, E2 also can be synthesized from testosterone by CYP19 activity in one step [18,19,20]. Due to the fact that E2 is lipophilic, it needs a transport molecule to pass through the aqueous phases of the intercellular space, so it binds to sex-hormone-binding globulins (SHBG) to reach the target cells [21,22].

According to the textbook knowledge, hydrophobic molecules can easily go through lipid membranes by simple diffusion [23]. However, in fact, the lipophilic nature explains why they are easily solved into the membrane, but it is hard to understand how they come out on the other side of the membrane. During the synthesis, even within the same cell, the intermediary metabolites go back and forth between the mitochondria and the smooth endoplasmic reticulum [24]. However, in the ovary, due to the lack of specific enzymes in numerous cell-types (e.g., granulosa cells do not have CYP17, while theca interna cells do not contain 17β hydroxysteroid dehydrogenase and CYP19), they are served between cells [25]. To ensure this, the membranes of the cooperating cells/cell organelles have to be rather close, and some chaperon molecules (e.g., heat shock proteins [23] and internalized SHBG [26]) should catch the molecules on the other side of the membrane. Indeed, ex vivo dissolved organic material can bind estrogens, although their aim is to eliminate them from the system [27].

### 2.2. Estrogen Receptors and Their Localization

**Figure 4:**
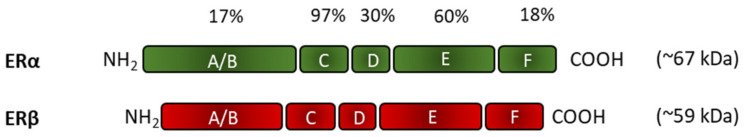
Structural units, homology, and size differences of estrogen receptors. A/B: NH2-terminal domain; C: DNA-linker domain; D/E/F: ligand-binding domain. The percentage indicates the degree of amino acid sequence homology between the different unites of ERα and ERβ.

Jensen and Jacobsen concluded 60 years ago that the E2-induced biological effects in the uterus are due to a specific protein [28]. In 1986, two independent research teams cloned this receptor from the uterus, which was later named estrogen receptor alpha (ERα) [29,30]. The development of genetic engineering and studies on ERα knock out (KO) mice have helped the discovery of another estrogen receptor, named ER beta (ERβ) [31,32,33,34]. ERα and ERβ are members of the steroid/thyroid hormone nuclear receptor superfamily with a common structure [35,36,37]. They have three independent but interacting domains: the NH_2_-terminal or A/B domain, the C or DNA-linker domain, and the D/E/F or ligand-binding domain. ERα and ERβ have differences in their ligand-binding domain and their size: ERα is larger (approx. 67 kDa) than ERβ (approx. 59 kDa) [38,39,40] (Figure 4).

Moreover, alternative splice variants may further diversify the localization and function of the receptors [41]. For example, the originally described ERβ (ERβ1) has high affinity for E2 and is localized in the nucleus. In contrast, ERβ2 contains an 18 amino acid insert within the ligand-binding domain and has low affinity to E2. The ERβdelta3 variant—due to a deletion in exon 3—does not bind to the estrogen response element in DNA, while the ERβdelta4 variant—due to another deletion in exon 4—is localized to the cytoplasm. 

Even though ERs are present in almost all cells of the body, ERα and ERβ show a large difference in their expression pattern. ERα mRNA is highly expressed in the epididymis, testes, ovaries, breasts, pituitary gland, uterus, kidneys, bones, and adrenal glands, while ERβ is detectable in the ovaries, colon, vascular endothelium, and prostate. In addition, the testes, uterus, bladder, and lungs show only moderate expression of ERβ mRNA [38]. Both ERα and ERβ is present in the central nervous system (CNS), but also with distinct expression patterns [42]. ERs are mostly present in the cytoplasm and after ligand binding they are translocated to the nucleus (cytonuclear receptor [43]). Interestingly, approximately 10% of the ERs are also found to be extra-nuclear in lipid-rich membrane organelles—also called membrane estrogen receptors 1 or mESR1 [44]— including cell-membrane lipid rafts, but also mitochondria, endoplasmic reticulum, and cytosolic endosomes [45]. Membrane-located classical ERs are connected to scaffolding proteins, like caveolin-1 [7,46,47] and considered to be alternative sex steroid receptors. Interestingly, there is a smaller isoform, the ERα36, which exists as an independent receptor and can be found presumably in the membrane [48]. Next to the classical receptors, we must mention the GPR30, or by its new name: GPER1 (G-protein-coupled estrogen receptor 1) [49]. This is a G-protein-coupled membrane receptor with seven transmembrane domains. In vitro studies in the early 2000s showed that E2 can activate this receptor, and it is responsible for the non-classical estrogen actions (see Section 3.2) [50,51,52,53]. GPER1 is highly expressed in the central and peripheral nervous system, ovaries, uterus, mammary glands, gastrointestinal system, bone tissue, cardiovascular system, immune cells, and adrenal glands [52,54,55,56]. 

## 3. Mechanism of Action

### 3.1. Classical Estrogen Actions

**Figure 5:**
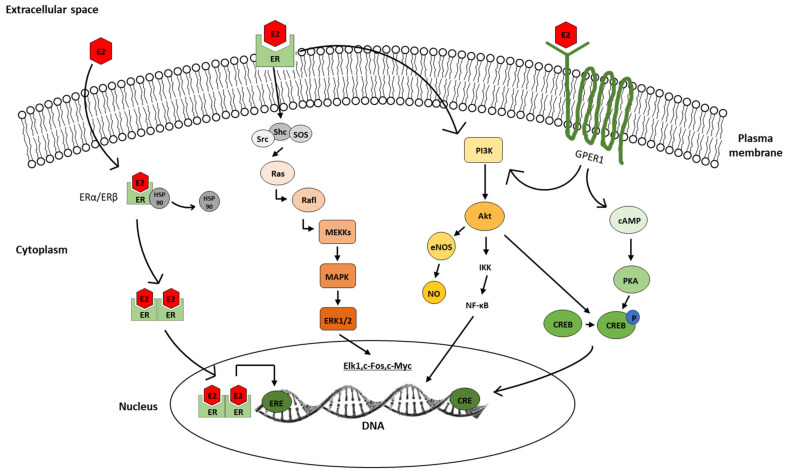
The cellular mechanisms of E2 action. Representative figure depicting the classical and non-classical pathways, including second messenger molecules and downstream signaling. Abbreviations: estradiol (E2), estrogen receptor (ER), heat shock protein 90 (HSP90), estrogen-responsive element (ERE), non-receptor tyrosine kinase (Src), src homology 2 domain-containing transforming protein (Shc), son of sevenless (SOS), RAS GTPase protein (Ras), RAF kinase (Rafl), mitogen-activated protein kinase kinase (MEKKs), mitogen-activated protein kinase (MAPK), extracellular signal-regulated kinases 1/2(ERK1/2), elk-1 transcription factor (Elk1), c-Fos transcription factor (c-Fos), MYC proto-oncogene, BHLH transcription factor (c-Myc), phosphatidylinositol-3-OH kinase (PI3K), protein kinase B (Akt), IκB kinase (IKK), nuclear factor kappa-light-chain-enhancer of activated B cells (NF-κB), endothelial nitric oxide synthase (eNOS), nitric oxide (NO), G-protein-coupled estrogen receptor (GPER1), cyclic adenosine monophosphate (cAMP), cAMP-responsive element-binding protein (CREB), adenylate cyclase (AC), protein kinase A (PKA), cAMP-responsive element (CRE).

E2 is a lipophilic molecule and, therefore, passes through the cell membrane by passive diffusion, and in the cytoplasm, it binds to intracellular estrogen receptors [57]. The biologically active ligand–receptor complex induces a complicated sequence of events that begins with conformational changes in the receptor, followed by receptor dimerization, and leads to direct translocation of the activated ligand–receptor complex to the nucleus [58,59]. This process is promoted by several chaperon molecules including heat hock protein 90 (HSP90) [60]. The dimerized receptor complex interacts with several nuclear receptor coactivators, such as the 160-KD steroid receptor coactivator protein (P160) or the cyclic adenosine 3′, 5′-monophosphate (cAMP)-responsive element-binding protein (CREB) [61,62,63]. The entire complex then binds to a specific DNA sequence, the estrogen-responsive element (ERE), to stimulate transcription, which ultimately leads to the synthesis of new proteins [64,65] (Figure 5). Cytonuclear ERs are key receptors in activating the classical pathway. However, the classical mechanism of action alone is not sufficient to fully elucidate the broad spectrum of estrogenic effects.

### 3.2. Non-Classical Estrogen Actions

The non-classical mechanism of E2 is mediated by direct effects on ion channels or by the activation of intracellular signaling via protein kinase A (PKA), phosphatidylinositol-3-OH kinase (PI3K), and mitogen-activated protein kinase (MAPK) pathways [66,67] (Figure 5). E2 can directly bind to membrane receptors and rapidly activates intracellular signaling pathways, leading to ERE-independent indirect gene transcription [68,69]. In addition to initiating transcription of non-ERE-dependent genes, another key feature of the non-classical pathway is the speed of response. An early study by Szego and Davis showed a rapid increase in cAMP levels in uterine tissue, 15 s after E2 administration [70]. Given that cAMP is a secondary messenger molecule of intracellular signaling, this study also aimed to provide evidence for the involvement of the PKA pathway in rapid E2 signaling. In addition, E2 has been reported to activate the MAPK pathway in seconds [71]. Firstly, the non-receptor tyrosine kinase (Src), Src homology 2 domain-containing transforming protein (Shc), and son of sevenless (SOS) complex activate Ras GTPase. Secondly, the complex binds to Raf and through a phosphorylation cascade on mitogen-activated protein kinase kinase (MEKKs) and MAPK, the extracellular regulated kinase ½ (ERK1/2) will modulate the transcription of genes [72]. An alternative pathway is via PI3K signaling molecules that can be activated by ERs as well as by GPERs [67,70,73,74] and interact with Akt, resulting in the activation of endothelial nitric oxide synthase (eNOS) [75,76,77,78,79,80,81].

One of the most important endpoints in the non-classical estrogen signaling is the cAMP-responsive element-binding protein (CREB). Phosphorylated CREB can form a homodimer or heterodimer with the cAMP-responsive element modulator (CREM) or can activate transcription factor (ATF), which then binds to the cAMP-responsive element (CRE) on the DNA [82,83,84]. Ca^2+^ plays a pivotal role in the activation of PKA, ERK1/2, and CREB [83]. Electrophysiological and Ca^2+^ imaging measurements showed that E2 can increase intracellular Ca^2+^ levels, which also explains the activation of PKA-ERK1/2-CREB signaling systems [85,86].

## 4. The Problem with E2 and Hormone Replacement Therapy (HRT)

Nowadays, E2 therapy is used in hormonal contraception, HRT, and in feminizing hormone therapy to treat gender dysphoria in transgender women [87]. Indications for the use of HRT in the postmenopausal age have risen sharply since the late 1980s, claiming that “menopause is a hormone deficiency disease, curable and totally preventable, just take estrogen” [88], as several epidemiological studies have shown that the use of HRT reduces the risk of osteoporosis, coronary heart disease, and all-cause mortality [89,90]. However, large-scale, controlled clinical trials in the late 1990s have resulted in a reassessment of the favorable paradigm of HRT. In 1998, the Heart and Estrogen/progestin Replacement Study (HERS) showed that HRT did not reduce the risk of recurrent myocardial infarction [91]. Subsequently, in 2002 and 2004, the Woman Health Initiatives (WHI) clinical studies showed that neither the estrogen–progesterone combination, nor the estrogen alone reduced the risk of coronary heart disease [92,93,94]. Moreover, the combined HRT even increased the risk of stroke, venous thromboembolism, and breast cancer. Therefore, HRT is no longer recommended for prevention of either coronary artery disease or osteoporosis, and the beneficial effects are neglected due to these risk factors and severe side effects. A recent 20-year follow-up of the WHI study confirmed that in hysterectomized women, conjugated equine estrogen (CEE) alone may decrease the prevalence and mortality of breast cancer; however, in women with intact uterus, CEE in combination with progesterone may increase its risk [95]. Thus, there is no “one size fits all” solution; individual differences should be taken into consideration also by HRT.

In addition to its cardiovascular and endometrial adverse effects, HRT may also have a negative impact on dementia development, although the question is still controversial. The higher prevalence of dementia, including Alzheimer’s disease (AD), in females, especially in postmenopausal women, suggests a strong correlation between E2 and memory function [96,97,98]. Indeed, E2 is involved in wide range of brain functions, from neuronal development to neuronal plasticity and memory formation [99,100]. Current research has shown positive effects [101,102,103] or ineffectiveness of HRT in cognitive disorders [104]; however, the Women’s Health Initiative Memory Study (WHIMS) provided evidence for worsening dementia [105,106,107,108]. All in all, it was concluded that HRT may reduce the risk during the first five years of menopause, but its long-term use may increase the risk of AD. Interestingly, in a recent report, three days of E2 treatment enhanced core aspects of creativity and verbal memory in young male individuals [109], supporting gender-independent positive effects.

The serious side effects steered estrogen research into a different direction. The common goal is to find new formulas or molecules that do not have the harmful side effects of estrogen but have a positive effect on menopausal symptoms, including nervous system problems, vascular lesions, and osteoporosis.

## 5. Possible New Therapies: SERMs, Phytoestrogens, SERDs, and ANGELS

Several estrogen-like compounds are available with wide therapeutic indications, divergently influencing different estrogen-related pathways. 

### 5.1. Selective Estrogen-Receptor Modulators (SERMs)

**Figure 6:**
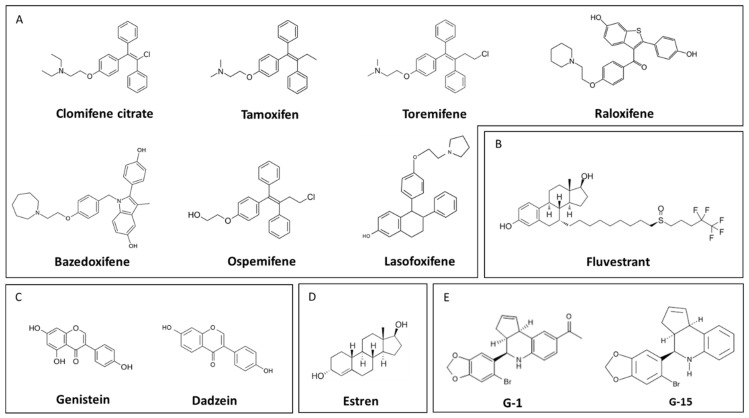
Representative structure of different estrogen-receptor modifiers. (**A**) Selective estrogen-receptor modulators (SERMs), (**B**) selective estrogen-receptor downregulator (SERD), (**C**) phytoestrogens, (**D**) activators of non-genomic estrogen-like signaling (ANGELS), (**E**) G-protein-coupled estrogen receptor 1 (GPER1) agonist (G-1), and GPER1 receptor antagonist (G-15).

Selective estrogen-receptor modulators (SERMs) are a group of nonsteroid compounds that have tissue-selective mechanisms of action (Figure 6A). SERMs act as partial estrogen receptor agonists in one (e.g., bone tissue), and antagonists in other (e.g., breast cells) tissues. In 2000, four compounds from the SERM and antiestrogen families were in clinical use in the United States (US): clomiphene, tamoxifen (TMX), toremifene, and raloxifene (RLX) [110]. The clinical database on these compounds is among the largest available.

Clomiphene citrate was the first molecule with estrogenic action developed for the treatment of polycystic ovarian syndrome (POS) and has been in use to treat female infertility since 1967 [111]. Originally, it was known as a competitive antagonist of E2 at the cytoplasmic nuclear receptor, but later it was considered as the first compound in the SERM family. In case of the reproductive system, it acts centrally, modulating—among others—the pulsatile activity of gonadotropin-releasing hormone (GnRH) neurons by blocking the negative feedback of E2. However, it can be used in the treatment of male hypogonadism and gynecomastia as well [112]. In bones, clomiphene seems to have an estrogen-like effect, preventing osteoporosis with similar efficacy as E2 in animal models [113]. On the other hand, in testosterone-deficient men younger than 50 years, it further decreased bone mineral density [114].

TMX was the first SERM developed for the treatment of ER-positive breast cancer [115]. It is on the World Health Organization (WHO) “List of Essential Medicines” [116], and in 2018, it was prescribed more than 1 million times in US [117]. In MCF-7 human breast adenocarcinoma cells, TMX slowed down cell proliferation, confirming its antiestrogenic potential [118]. Indeed, TMX (20–40 mg/day) treatment improved the 10-year survival of women with ER-positive breast cancer and reduced breast cancer reassurance in several clinical trials [119,120]. Moreover, similarly to clomiphene citrate, TMX can be an ovulation initiator through the stimulation of the secretion of follicle-stimulating hormone (FSH) at the pituitary [121]. On the other hand, TMX is an agonist in the bones, protecting bone integrity via inhibition of the osteoclast activity in vitro and in vivo in postmenopausal women [122,123]. However, it may have an opposite effect on bone mineral density (i.e., promoting bone loss) in premenopausal women, depending on the menstrual status [124,125]. TMX is an ER agonist in the cardiovascular system; it decreases low-density lipoprotein (LDL) (“bad” lipoprotein) concentrations and it increases the amount of coagulation factors, and thus, the risk of thrombosis and stroke [126]. In the brain, TMX might have neuroprotective effects by activating the astrocytes and microglia after brain trauma or injury [127,128,129]. Unfortunately, TMX is an agonist on the endometrial ERs as well. Thus, TMX-induced endometrium proliferation may increase the risk of endometrial carcinoma [130]. 

Toremifene is a second-generation SERM, with very similar biological effects as TMX [131]. It can be used in ER-positive breast cancer with the same efficacy, but with a better safety profile [132,133].

RLX is also a second-generation SERM, currently used to treat osteoporosis. In 1999, a 3-year randomized placebo-controlled study named Multiple Outcomes of Raloxifene Evaluation (MORE) revealed that RLX (60–120 mg/day) increased bone mineral density and reduced the vertebral fractures in postmenopausal women [134]. The study also showed that RLX has an antagonist effect in the breast and reduces breast cancer prevalence by 76% [135]. Therefore, RLX is often used to treat osteoporosis in patients with high risk of ER-positive breast cancer [136]. In the cardiovascular system, although RLX decreases LDL cholesterol levels, it has the same effect on nitric-oxide (NO) formation as E2, and thus, increases the risk of thrombosis and stroke. However, compared to E2, TMX, and toremifene, RLX lacks endometrial side effects. 

Bazedoxifene (BZX) is a third-generation SERM used for osteoporosis treatment, with a unique mechanism of action. In MCF-7 cells and animal models of breast cancer, BZX increased the degradation of ERα by inducing a conformational change on the receptor [137,138]. This suggests an E2-antagonistic effect. All in all, BZX lacks breast and endometrial side effects, and thereby has an improved safety profile [139]. BZX crosses the blood–brain barrier (BBB), and enhances spatial memory in OVX rats, a model of postmenopausal estrogen decline [140]. 

Ospemifene is another third-generation SERM, currently used to treat the genitourinary syndrome of menopause (GSM, see Section 6.2.2.3) [141]. Being a partial agonist in the endometrium, it has positive effects on vaginal dryness and atrophy [142]. As an agonist in bones, it is advantageous in osteoporosis. However, it may exert estrogenic side effects in the vascular system [143]. 

Lasofoxifene (LFX), from the same generation as BZX and ospemifene, is used in osteoporosis prevention and to treat vaginal atrophy. The Postmenopausal Evaluation and Risk Reduction with Lasofoxifene (PEARL) study showed that LFX improves bone mineral density, and reduces the prevalence of vertebral fracture by 42% [144]. As a receptor antagonist in breast, it reduced the risk of ER-positive breast cancer by 81% [144]. An increased risk of venous thromboembolism was detected, but no increase in other coronary events or stroke [145].

Thus, newer SERMs have favorable E2-antagonistic and -agonistic profiles with enhanced efficacy and less side-effects. 

### 5.2. Phytoestrogens

Phytoestrogens are plant-based estrogen-like substances. Their mechanism of action is comparable to SERMs, because they have tissue-specific antagonist and agonist properties. Their effect might depend on E2 concentrations [146]. Isoflavonoids may act as estrogen antagonists with the premenopausal concentration of E2, whereas they exert estrogen agonistic activity with lower E2 concentration, close to the serum levels of postmenopausal women. Genistein and daidzein are the two major compounds isolated from soybeans, and belong to the group of isoflavones [147] (Figure 6C). 

Several articles showed that phytoestrogens have a positive effect on post-menopausal women [148]. Genistein supplements reduced menopausal symptoms, such as hot flushes [149,150]. Phytoestrogens are also agonists in the bone tissue; therefore, they have a positive influence on osteoporosis [151]. Daidzein stimulates MAPK/PI3K pathways in bones, exhibiting bone-sparing effects against osteoporosis [152]. A mixture of genistein, daidzein, and equol, which has ERβ binding selectivity, proved to be neuroprotective and promoted estrogenic mechanisms in the brain, while avoiding feminizing activity in the reproductive system [153,154]. Phytoestrogens are also anticarcinogenic in the breast, by exerting antiangiogenic, antimetastatic, and epigenetic effects [155]. In OVX rats, genistein shows a similar vasodilative, vasoprotective effect as E2 treatment and increases NOS activity without affecting the mass of the uterus, and thus, lacks endometrial side-effects [156]. In another experiment, it was shown that genistein (and RLX) ex vivo increased NOS activity and decreased NO-mediated platelet aggregation [157]. Thus, its cardiovascular profile seems to be also beneficial.

### 5.3. Selective Estrogen-Receptor Downregulators (SERDs)

Selective estrogen-receptor downregulators or degraders (SERDs) are a group of molecules with the ability to downregulate classical estrogen receptors [158]. In fact, the previously mentioned BZX also has some similar effect. Fulvestrant was developed as a steroidal antiestrogen and currently it is the most important molecule from the SERD group (Figure 6B), which binds to ERs in a monomeric form, and inhibits dimerization [159]. It reduces the half-life of the receptor protein without influencing its mRNA levels [160]. Fulvestrant is currently in use to treat advanced ER-positive breast cancer, but also showed a minimal positive effect in a Phase II endometrial cancer study [161,162].

Fulvestrant is used as an intramuscular injection, but different oral SERD agents are also under investigation [163], especially for the therapy of treatment-resistant breast cancer [164]. Indeed, several non-steroidal molecules such as RAD-1901, AZD-9496, GDC-0927, etc. are already in clinical trials.

### 5.4. Activators of Non-Genomic Estrogen-like Signaling (ANGELS)

Activators of non-genomic estrogen-like signaling (ANGELS) are a new approach in E2 therapy. ANGELS can selectively activate the non-classical action of E2 [165]. There are three known molecules with such effect: estren (4-estren-3alpha, 17beta-diol), compound A (2-(4-hydroxyphenyl)-3-methylbenzo[b]thiophen-5-ol), and compound B (3′,15b-dihydrocyclopropa [14,15]estra-1,3,5(10),8-tetraene-3,17alpha-diol), but neither of them are used in therapy yet [166,167,168]. 

Estren has bone-protective effects (see Section 6.2.3.1) without influencing endometrial proliferation [167] (Figure 6D). It was shown to have neuroprotective potentials on basal forebrain cholinergic (BFC) neurons in an animal model of AD [169,170]. Moreover, estren also induces vasodilatation and has vasoprotective effects (see Section 6.2.2.1).

Specific GPER1 agonists and antagonists may also influence the non-classical estrogenic mechanism of action. G-1 is a GPER1 receptor agonist that mediates rapid signaling [171] (Figure 6E). G-1 was proven to be effective in preclinical studies regarding multiple sclerosis, ischemia, stroke, epilepsy, and brain injury [172,173,174]. However, in murine bone marrow mesenchymal stem cells and endometrium cells, G-1 had proliferative properties [175,176]. Therefore, G-1-like compounds may possibly have carcinogenic side-effects. In contrast, GPER1 antagonists such as G-15 and G-36 [177,178] have anticarcinogenic properties [176] and might be used to fight cancer (Figure 6E).

Beside their therapeutic potential, novel HRTs and potential therapeutic agents (see Table 1 for summary) help us to better understand the details of physiological E2 action. The perfect therapy with neuroprotective, antithrombotic, osteoprotective, and anticarcinogenic effects is yet to be discovered, but accumulating data might bring us closer to it. 

### 5.5. Aromatase Inhibitors

Beside manipulating its receptor, another possibility to modulate the E2 level is to interact with the synthesizing enzymes. Indeed, CYP19 (aromatase) is a crucial step in the E2 synthesis (see Figure 3), and its activity can be pharmacologically inhibited by aromatase inhibitors [179]. This might be beneficial when enhanced E2 levels are harmful, especially in the case of E2-dependent cancers. Testing other possible indications, such as hypogonadism, did not lead to satisfactory results [180]. In this case, for example, SERMs were more beneficial.

Interestingly, some phytoestrogens, such as genistein—beside interacting with ERs, see Section 5.2—may even stimulate aromatase [181]. 

Nevertheless, E2 replacement rather than reduced E2 levels is desirable in most cases, and selective, tissue-specific interventions (such as SERMs and ANGELS) may provide such targeted manipulation more satisfactorily.

### 5.6. Future Direction of HRT

Due to its multidimensional beneficial effect, HRT was widely introduced, but large-scale studies highlighted its side-effects (see Section 4). Therefore, although HRT is still a standard therapy in women with premature ovarian failure (age bellow 51) for the prevention of osteoporotic complications, its usage for menopausal hormone therapy is still controversial [182]. Nevertheless, new guidelines recommend its usage for Vasomotor symptoms (VMS) and vulvo-vaginal atrophy (GSM) in women aged less than 60 years or who have had menopause for less than 10 years [183].

## 6. Estrogen Effects with Therapeutic Consequences

### 6.1. Main Role of E2 on Reproduction; Contraceptives

The hypothalamic–pituitary–gonadal axis (HPG) is the most important endocrine pathway in the regulation of reproduction. Neuroendocrine control of the reproductive axis is performed directly by the GnRH-producing neurons, scattered in the basal forebrain [184] with a prominent occurrence in the preoptic area of the hypothalamus (POA) in humans [185]. GnRH production stimulates the release of luteinizing hormone (LH) and FSH in adenohypophysis, of which FSH stimulates estrogen secretion in the granulosa cells of the ovary [186,187,188]. 

**Figure 7:**
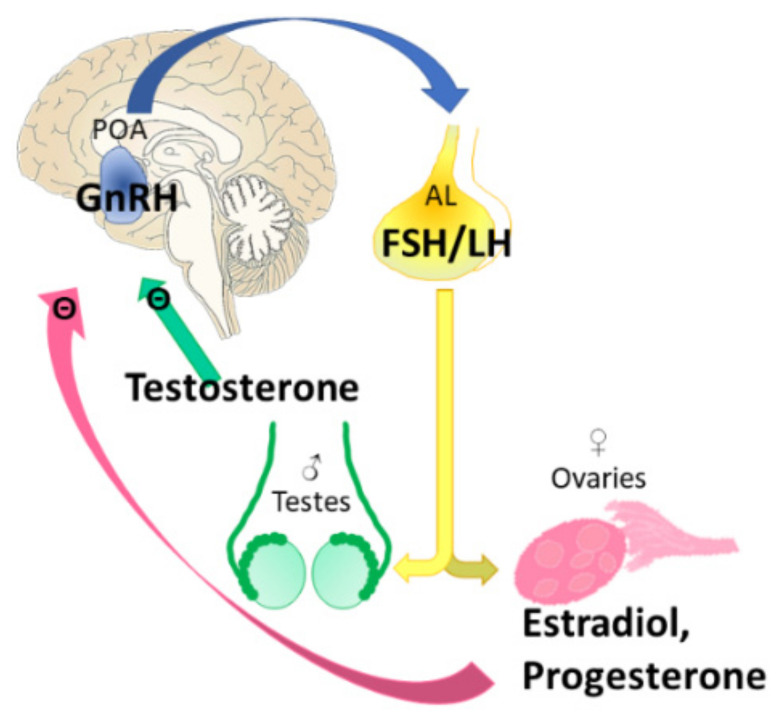
Hypothalamic–pituitary–gonadal axis (HPG). The gonadotropin-releasing hormone (GnRH)-producing cells are mainly present in the preoptic area (POA) and reach the anterior lobe of the pituitary (AL) through the hypophyseal portal circulation, stimulating the synthesis and release of follicle-stimulating hormone (FSH) and luteinizing hormone (LH), which, through the general circulation, affect the target organ (ovaries in females, while testes in males). The end hormones estrogen/progesterone and testosterone provide negative feedback.

The most critical point of this reproductive communication system is the estrogen-dependent regulation of GnRH. The pulsatile activity of the GnRH neurons is attributable to the negative feedback of E2. In the brain of male rodents, E2 is produced by the CYP19 (aromatase) enzyme from testosterone. This mechanism exerts a constant inhibition on the pulsatility of GnRH neurons [189,190]. In females, during the majority of the estrus cycle, GnRH is under E2 negative feedback too [191] (Figure 7).

In 2012, István Ábrahám and colleagues explored this mechanism in mice, and found that knocking out the CREB in GnRH neurons resulted in abnormal E2 negative feedback. Therefore, they concluded that rapid, non-classical action of E2 on CREB phosphorylation may be an important player in the estrus cycle regulation, at least in mice [192]. Indeed, it was previously described that the non-classical pathway might be involved in E2’s negative feedback actions at the level of the hypothalamus and/or pituitary gland [68]. Besides, the same study showed that the positive feedback of E2 during the LH-surge, inducing ovulation [193,194], is regulated by classical ERα signaling [68,195]. The lordosis behavior, observed in female rodents as the sign of sexual receptivity, is also influenced by rapid E2 actions through membrane signaling on neuropeptide Y and µ-opioid receptor circuits [196]. However, E2 not only regulates ovulation but also plays a role in other processes during the menstrual cycle. For example, during endometrial regeneration, E2 supports angiogenesis presumably via ERβ [197]. Furthermore, E2 is one of the key hormones in the regulation of human parturition due to its inducing of changes in the uterus and cervix, which are crucial for labor and birth (for a detailed review, see [198]).

The role of GPER1, as a main player in non-classical E2 actions in the reproductive system, is widely studied. However, investigations in humans are still rare [199]. GPER1 expression, among many other tissues, can be found in endometrium during the proliferative phase, where it might contribute to estrus cycle regulation via rapid, non-classical actions [200]. GPER1 agonist and antagonist treatments have suggested that this receptor subtype may directly affect estrogen-induced epithelial proliferation in mice uterus [177]. In a study with another rodent species, GPER1 expression in ovarian granulosa and in theca cells was found to be regulated by gonadotropins (FSH and LH) [201]. Moreover, the same research group described that GPER1 regulates the E2-mediated stimulation of primordial follicular formation in hamsters [202]. A recent study in mice examined herbicide-induced oocyte maturity failure, and found an activation of GPER1 dysregulation as a possible cause. The herbicide-disrupted oocyte maturation was rescued by a GPER1 receptor antagonist [203]. This result suggests that non-classical estrogen action through GPER1 may be present in oocytes not only in lower vertebrates but also in mammals. Indeed, this receptor subtype has been described in the mouse oocyte membrane during maturation [204] as well as in human granulosa cells, where its expression was induced by LH [205]. Additionally, in a human study, GPER1 activation was found to potentiate the contraction response of the myometrium to oxytocin [206]. Based on this finding, the role of this G-protein-coupled receptor can be assumed during labor.

**Figure 8:**
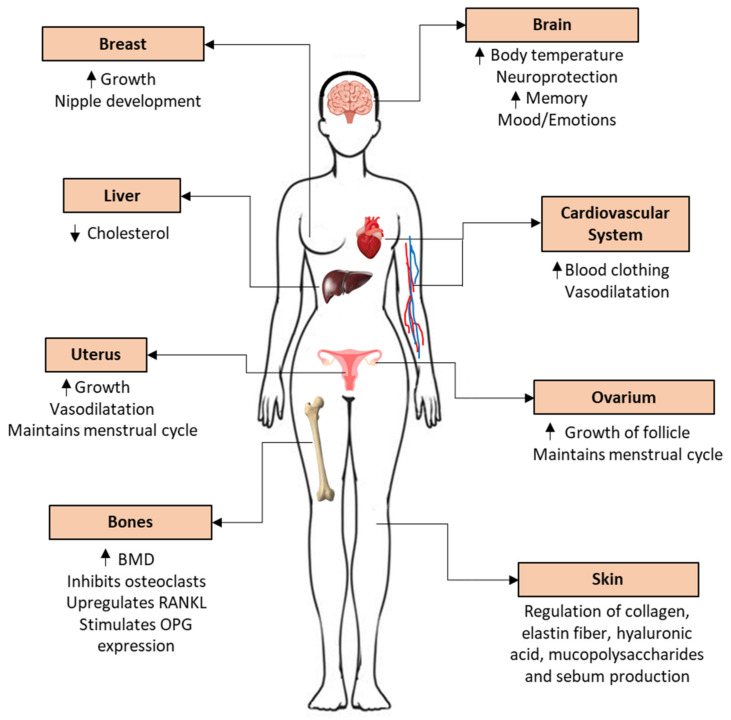
Estrogen effects throughout the body and the role of E2 in a tissue-specific manner. Up arrow—increase; down arrow—decrease. Abbreviations: bone mineral density (BMD), receptor activator of nuclear factor kappa-Β ligand (RANKL), osteoprotegerin (OPG).

Due to its widespread role in reproduction via classical as well as non-classical signalization, estrogen is often used in contraception, mostly in combinations with progesterone, in many forms from oral pills, transdermal patches, and vaginal rings to contraceptive coils (Figure 8). 

As a contraceptive agent, the main mechanism of E2 is the negative feedback on GnRH neurons discussed above [187] (Figure 7). Hormonal contraception—similarly to HRT (see Section 4)—has many contraindications, because it can increase thromboembolic disorders such as stroke and acute myocardial infarct, and increase the risk of cancer development in exposed women [207]. Therefore, previously mentioned alternative substances may be eligible. Indeed, estetrol (E4), a natural human estrogen produced during human pregnancy in the fetal liver, behaves as a natural SERM, and was approved in 2021 in Canada in combination (with drospirenon, a progestin medication) as an oral contraceptive [141,208]. E4 acts as an estrogen agonist on the vagina, uterus, and endometrium, and also shows bone-sparing activity. Moreover, in a mouse model, it had a favorable cardiovascular effect with antithrombotic activity [209]. On the contrary, other SERMs with antiestrogen activity may normalize amenorrhea and restore reproduction, especially in POS (see Section 5.1 Clomiphene citrate, Table 1).

### 6.2. Menopausal Symptoms

Menopause affects every woman at a certain age by influencing their quality of life and their performance at work [210]. Premenopause is also associated with greater hormone fluctuations compared to the luteal and follicular phases of the menstrual cycle, which may cause severe neurochemical changes in the CNS, leading to emotional disturbances as well as cognitive alterations [211]. Menstruation is permanently lost due to loss of ovarian follicular activity, leading to a gradual decrease in circulating E2 during the menopausal transition. In postmenopause, E2 stabilizes at a constant low level [212]. The main symptoms of this period are as follows: hot flushes, mood changes, memory deterioration, migraine, metabolic and cardiovascular changes, skin and hair aging, muscle degradation, and osteoporosis.

#### 6.2.1. Effects in the Brain

##### 6.2.1.1. Hot Flushes

Hot flushes affect 75% of menopausal women [211]. They occur mainly during the first stages of sleep, influencing its quality [213]. Decreases in E2 levels at the thermoregulatory center of the hypothalamus influence the activity of catecholamine and noradrenergic pathways (i.e., sympathetic activity), ending in vasomotor symptoms (VMS) [214] (Figure 8). Thus, CNS changes are the source of hot flushes, and mainly the thermoregulatory centrum in the POA is affected [215]. In addition, hypertrophy of ERα-expressing neurons in the infundibular nucleus is also characteristic, with increased kisspeptin, neurokinin B, and substance P mRNA concentrations [216,217,218,219]. Moreover, at the level of the pituitary, the withdrawal of ovarian estrogen-dependent negative feedback during menopause leads to increased LH synthesis [220] (Figure 7). Changes in the expression pattern of these genes lead to increased signaling to heat dissipation effectors in the CNS [221].

Due to detrimental side effects, HRT is not recommended nowadays (see Section 4). Even some widely used SERM, such as TMX [222] or the third-generation ospemifene [223,224], might induce and not ameliorate hot flushes. Thus, for the treatment of hot flushes, phytoestrogens are widely used, being closer to something “natural” (Table 1). Indeed, in a recent study, nutraceuticals containing—among others—soy isoflavone significantly reduced both hot flushes and sweating during a 6- and 12-week treatment period [225]. Another study also found significant improvement of the menopausal symptoms reflected by the Kupperman Index (an 11-item menopausal symptom questionnaire) after 90-day phytoestrogen treatment [226]. A study based upon self-reports of several thousand women concluded that soy products but not soy milk are associated with lower likelihood of reporting subsequent VMS [227]. It was also suggested that genistein, an important isoflavone of soy-derived food, may reduce hot flushes via the inhibition of visfatin, an inflammatory adipokine [228].

##### 6.2.1.2. Mood Swings

Female sexual hormones deeply influence mood, as reflected by cyclic changes during the menstrual cycle, and especially by dysphoria during the premenstrual phase [229]. A recent meta-analysis showed an approx. 40% prevalence of premenstrual syndromes in Indian women [230]. Moreover, ongoing mood disorders may also be exacerbated during the premenstrual period [231]. Additionally, there is a well-known sex difference in the prevalence of anxiety and depression with higher occurrence in females. COVID-19 pandemic-induced depression is also more prevalent in women [232], and functional gastrointestinal disorders, such as irritable bowel disease, are also more common in them [233]. There is a female preponderance of adolescent insomnia [234], which may also contribute to mood alterations. Moreover, sex hormones differentially organize dimorphic circuits during sensitive developmental periods [235]. All of this suggests that mood stands under strong influence of sexual hormones, presumably under the influence of E2. Indeed, there are ERs in the amygdala [236,237] and hippocampus, parts of the limbic system that are well-known for their regulatory roles in emotions. However, the hippocampus is important for spatial memory formation, while the amygdala is also important in social behavior regulating, e.g., pair bonding and dominance through ERα [236,237]. The amygdala was also shown to have non-classical, CREB-related E2 signaling [238]. 

Anxiety and depression are stress-related disorders and highly connected with stress-regulation disturbances (among others, alteration in the hypothalamic–pituitary–adrenocortical axis (HPA)). E2 fluctuation during menopause may contribute to dysregulation of the HPA axis, leading to perimenopausal depression [239]. 

All in all, these E2-deficiency-induced anxiety- and depression-related symptoms can be treated by E2, and preferably with newer, non-classical E2-like substances.

##### 6.2.1.3. Neuroprotection

The higher prevalence of dementia in postmenopausal women suggests that female sexual steroids, especially E2, might be preventative [240]. Indeed, there are significant amounts of ERs in the centers of episodic and working memory, the hippocampus and prefrontal cortex, so during perimenopause, the E2 fluctuation can cause a significant cognitive deficit [241]. However, besides classical pathways, non-classical signaling should be also taken into consideration. 

**Figure 9:**
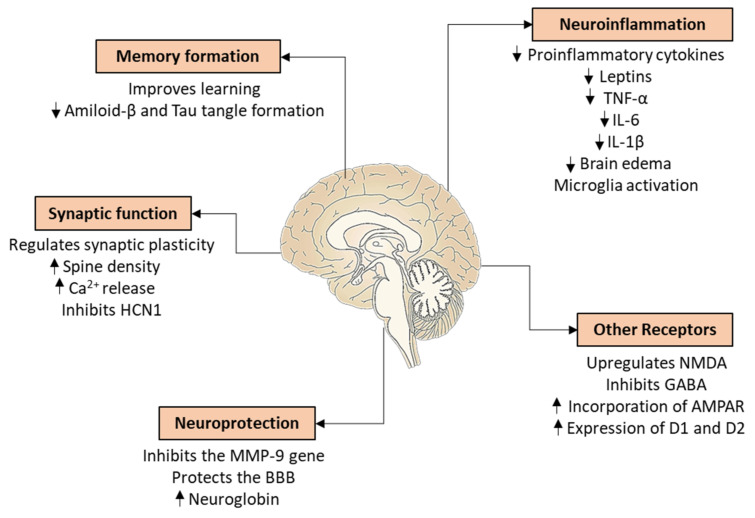
Neuroprotective effects of estrogens in the central nervous system. TNFα: tumor necrosis factor α; IL-6: interleukin-6; IL-1β: interleukin 1β; NMDA: N-methyl-D-aspartate; GABA: gamma-aminobutyric acid; AMPA: α-amino-3-hydroxy-5-methyl-4-isoxazolepropionic acid receptor; D1: dopamine receptor 1; D2: dopamine receptor 2; MMP-9: matrix metallopeptidase 9; BBB: blood–brain barrier; HCN1: hyperpolarization-activated cation channel. Up arrow—increase; down arrow—decrease.

Alongside memory formation, E2 also influences neuroinflammation (Figure 9). Unstable E2 levels in the brain were found to be associated with the development of neurogenic inflammation leading to migraine [242]. The connection is supported by the conclusion of the Prospective analysis of actors related to migraine aura (PAMINA) study, namely that the sudden decrease in E2 levels is a major risk factor for migraine [243].

The neuroprotective estrogen comes from different sources. Firstly, it comes from the periphery through the BBB. Secondly, it can be locally transformed from androsterone, an androgenic compound. Thirdly, E2 is produced directly from cholesterol by neurons or glial cells. All enzymes involved in estrogen biosynthesis are expressed in both neurons and astrocytes [244,245]. CYP19, which catalyzes the last biosynthetic step, is found in higher amounts in neurons than in glial cells [246]. The regulation of steroidogenesis in the brain is independent from the periphery, so the plasma steroid concentration does not correlate with the amounts measured in the brain [247,248]. However, GnRH, which regulates E2 synthesis in the ovary, also affects hippocampal production. During brain injury, astrocytes also begin to produce CYP19, and therefore E2 as a product. This seems to be essential for neuroprotection. There is an interplay between neurons and glial cells, as the activation of astrocytes depends on the E2 produced by the neurons [249,250]. 

Previous studies have shown the participation of the two most important receptors, ERα and ERβ, in the neuroprotective effects of E2 with a more prominent role of ERα [251]. E2 therapy also increased ERα expression during neuroinflammation [252]. On the other hand, ERβ was responsible for increasing dendritic spine density [253]. Moreover, E2-related neuroprotective mechanisms can also be mediated by GPER1. In a schizophrenia model of GPER1-KO mice, this receptor showed an important role in learning and memory [254]. In human BFC neurons—from the nucleus basalis of Meynert—both E2 and G-1 proved to have anti-inflammatory effects [56].

There are several possible mechanisms for the neuroprotective effect of E2. Firstly, it reduces the amount of proinflammatory cytokines, leptins, and also the expression of tumor necrosis factor α [255]. E2 therapy also decreased the interleukin-1β and interleukin-6 expression, and therefore decreased the incidence of brain edema in OVX female rats [256] (Figure 9). These processes may contribute to the reducing of neurodegeneration during neuroinflammation by reducing microglia activation. A further possible mechanism is the inhibition of the matrix metallopeptidase *9* (MMP-9) gene; in this way, E2 may protect the breakdown of the BBB after trauma [257,258] (Figure 9). Indeed, MMPs are important regulators of vascular and uterine remodeling, and are under the regulatory control of E2 and progesterone [259]. Furthermore, the level of MMP-9 is known to increase during stroke [260]. Next, E2 regulates synaptic function because ER activation may induce rapid alterations in neuronal excitability by means of the protein kinase-dependent phosphorylation of membrane ion channels. These effects depend on the type of neuron [261]. For example, E2 triggers increased Ca^2+^ oscillation through T-type channels and potentiates K^+^ channels in GnRH neurons. However, it reduces L-type and N-type Ca^2+^ current in neostriatal and cortical neurons with concomitant increases in excitability through fast K^+^ channels [262]. Moreover, E2 inhibits the hyperpolarization-activated cation channel (HCN1) in the hippocampal pyramidal and dorsal ganglionic neurons, an essential molecule for hippocampal information processing [262]. This process was mediated by GPER1 [263]. Another aspect of synaptic function is the dendritic spine density, which fluctuates in the hippocampus—but not in the cortex—during the estrus cycle [264]. However, removal of E2 by OVX decreased the spine density not only in the hippocampus, but also in the cortex, and this effect could have been prevented by HRT [265] (Figure 9). The same effects were observable in pyramidal cells in the sensorimotor cortex and medial nucleus of the amygdala in rats and in dorsolateral pyramidal cells of the prefrontal cortex in monkeys [266,267]. Additionally, E2—through GPER1—increased excitatory postsynaptic potentials in the entorhinal cortex of OVX rats, the major source of cortical sensory and associational input to the hippocampus [268]. Furthermore, E2 might alter the appearance and function of other receptors. In the hippocampus, E2 increased the excitability by upregulating the expression of N-methyl-D-aspartate (NMDA), an ionotropic excitatory glutamate receptor, in the plasma membrane. In parallel, E2 inhibited the inhibitory gamma-aminobutyric acid-erg (GABA) transmission, in part due to the interactions of ERα and a glutamate receptor, which led to the mobilization of anandamide, a retrograde signaling endocannabinoid [269]. E2 rapidly modulates synaptic plasticity including long-term potentiation (LTP) and depression [270]. Several steps lead to the development of LTP involving NMDA or metabotropic glutamate receptors, downstream phosphorylation cascades, dynamic changes in the postsynaptic membrane expression of α-amino-3-hydroxy-5-methyl-4-isoxazolepropionic acid (AMPA) receptors, another ionotropic glutamate receptor, CREB-mediated transcriptional activation, and microtubule-dependent transport processes; and E2 might influence most of them. For example, in the hippocampus, E2 promoted LTP by increasing the incorporation of the AMPA receptors into the postsynaptic membrane of pyramidal neurons [270]. On cultured cortical neurons, E2 transiently enhanced the internalization of AMPA, while it increased the incorporation of NMDA receptors into the plasma membrane. The work of our research group confirmed that E2, via non-classical actions mediated by GPER1, decreased the movement of AMPA receptors in neurites [271]. The glutamate receptor dynamics might be associated with rearrangement of the cytoskeleton by regulating actin polymerization and rearranging the morphology of the dendritic spine [272,273]. As an additional mechanism, E2 may modulate dopaminergic transmission in the nigrostriatal system through ERα and ERβ [274]. E2 acutely increased dopamine (DA) synthesis, release, and turnover, as well as the expression of the DA transporter and D1 and D2 receptors in the striatum [274]. E2 induced an increase in motor behavior not only due to an increase in DA release, but also by inhibiting the DA uptake in astrocytes [274]. Last but not least, E2 upregulates neuroglobin and translocates it to the mitochondria to sustain neuronal and glial cell adaptation to injury [275]. Indeed, neuroglobin may act as a gas sensor upon hypoxic insults and intervenes in anti-oxidant and anti-apoptotic signaling pathways [276]. 

Although the neuroprotective effect of E2 is obvious in females, similar effects can be detected in males as well. In this sense, ERβ was found to be involved in ischemia-reperfusion-induced cerebral damage in male rats [277]. Moreover, TMX, a SERM, prevented the ischemia-induced neuronal damage in the hippocampus of male rats [278,279]. Additionally, G-1 and G-15, a GPER1 agonist and antagonist, respectively, modulated synaptic plasticity during temporal lobe epilepsy in Sprague Dawley rats [173]. However, E2 modulated hippocampal neurogenesis and cell death in adult female rats, but not in male rats [280,281]. 

#### 6.2.2. Changes at the Periphery

##### 6.2.2.1. Cardiovascular Effects

The first clues on the regulatory role of E2 in the vascular system came from the observations of low incidences of coronary artery diseases in premenopausal women compared to men and postmenopausal women [282,283,284,285,286]. 

At first, it was thought to be due to the E2 effect on lipid homeostasis, but soon it was connected to its direct vascular effects [287], especially through the classical pathway induced by nuclear receptors. Endothelial and smooth muscle cells express nuclear ERα in the mammalian body [71,288,289]; thus, it was the first target. The role of ERα was further confirmed during vascular injury; however, there is a complex mechanism and interplay between splice variants resulting in this protective effect [290]. Interestingly, in smooth muscle cells, the activation of inducible NOS (iNOS) during inflammatory responses may lead to adverse vasodilatory effects; therefore, its termination is also important. E2 inhibits the over-activity of iNOS via ERα [291]. This protective function might be damaged under different pathological conditions, e.g., in diabetic rats [292]. ApoE KO mice are often used as an animal model for atherosclerosis with increased cardiovascular risk. In this strain, E2 treatment reduced OVX-induced atherosclerotic plaques and plasma cholesterol levels—at least partially—through the ERα [293].

On the other hand, ERβ is the main subtype occurring in the endothelial cells of the mammalian endometrium [78,294,295,296,297,298]; therefore, the cardiovascular E2 effects—having a pivotal role also in cyclic bleeding—were also connected to its activation. Although, in one study, ERβ expression showed a reactive response after vascular injury in male rats [299], other studies did not confirm this [76,300]. However, ERβ still seems to be connected to vasoprotective properties: in an in vitro human-brain vascular smooth muscle cell model, dihydrotestosterone, an androgen hormone, reduced inflammation via ERβ [301]. Another study showed that a specific ERβ agonist, indazole-Cl, also decreased inflammation after hypoxic stress [302]. Urocortin, an important player in stress adaptation, induced vasodilation also through ERβ; however, as of now, it is unknown whether this is regulated by genomic or non-genomic pathways [303]. Interestingly, secreted HSP27, an ERβ-associated protein, also has a positive cardiovascular effect promoting atheroprotection [304]. HSP27 levels were lower in plaques versus normal (disease-free) arteries and serum HSP27 levels were reduced in patients with stable coronary artery disease versus controls [304]. An ERβ-specific agonist, diarylpropionitrile, was able to reproduce the predicted atheroprotective effects of HSP27 [304]. Additionally, ERs may induce long-term changes, such as angiogenesis and vascular remodeling, to adapt to the physiological changes during pregnancy [79,305,306].

However, less is known about the membrane bound, non-classical pathways that exert rapid vasodilatation and, thus, may increase blood flow in the uterus [78,298,307]. Indeed, E2 can act in an ER-independent way in the smooth muscle cells, by inhibiting the Src pathway (see Figure 5) and, thus, the voltage-gated potassium channels that mediate serotonin-induced vasoconstriction [308]. On the other hand, other authors argued that this relaxant effect is mediated by the inhibition of L-type Ca^2+^ channels, and progesterone also plays a role in it [309]. Cerebral arteries are of particular importance where E2 regulates vasodilatation through the NO-NOS system [310]. In vitro and ex vivo experiments confirmed that—besides E2—ANGELS compounds (see Section 5.4) also induce vasorelaxation. However, they are doing so via both eNOS-dependent and -independent pathways [71,311,312,313], for which intact endothelia and arteries are required [314]. There is also evidence that the expression of insulin-like growth factor 1, a potent mitogen for vascular smooth muscle cells, is modulated by E2 without activating ERs [315]. Newer studies directly showed that GPER1 also plays a role in these processes [172,316]. In an in vitro study, E2 (in high concentrations) had relaxant effect on the contracting uteri of pregnant rats, which was mediated by non-genomic pathways and GPER1 [317].

While preclinical and cellular studies are promising, clinical studies show controversial results [91,318,319,320]. Although low-dose oral and transdermal HRT preparations were shown to be cardioprotective [321], overall HRT increased the risk of thromboembolic events rather than being beneficial (see Section 4). It is still controversial as to whether E2 agonists or antagonists would be beneficial. Therefore, a more selective compound without any effect on the cardiovascular system seems to be preferable (see Table 1). 

##### 6.2.2.2. Metabolic Disturbances

**Figure 10:**
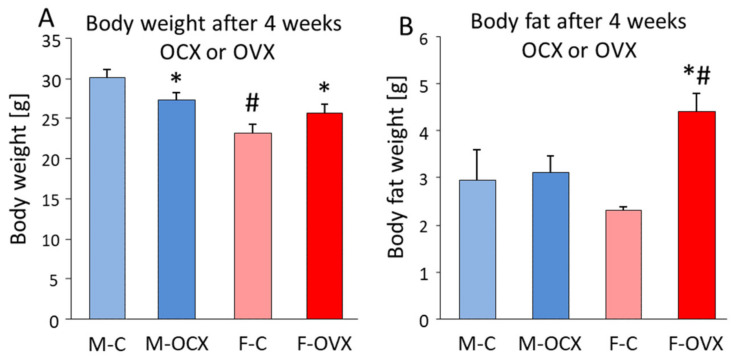
Effect of removal of sex hormones on the body composition in 4-month-old C57/Bl6 mice measured by magnetic resonance imaging (MRI) in the Metabolic Laboratory of the Institute of Experimental Medicine, Budapest, Hungary, 4 weeks after removal of the hormones (orchidectomy (OCX) in males and ovariectomy (OVX) in females under ketamine-xylazin anesthesia [327]). (**A**) The body weight difference showed significant sex difference (F_(1,34)_ = 39.5, *p* < 0.01) as well as sex * OCX-OXV interaction (F_(1,34)_ = 15.0, *p* < 0.01), with a decrease in males and an elevation in females. (**B**) For the body fat content, the removal of the sex hormones had a significant impact (F_(1,34)_ = 8.3, *p* < 0.01) in a highly sex-dependent way (interaction: F_(1,34)_ = 6.1, *p* = 0.02). More specifically, there was no change in males, and an increase in females. Abbreviations: M-C—male, control operated; M-OCX—male, orchiectomized; F-C—female, control operated; F-OVX—female, ovariectomized. * *p* < 0.01 vs. control operation of the same sex; # *p* < 0.01 vs. male.

One of the most disturbing changes around menopause is the weight gain leading to serious health problems. Indeed, postmenopausal women have higher rates of severe obesity when compared with their male counterparts [322]. Metabolic changes during menopause are due to a shift in E2 levels to an androgenic state [323]. The increase in bioavailable testosterone stimulates the accumulation of visceral fat, which is exacerbated by the lack of E2, further promoting the accumulation of centrally stored adipose tissue [324,325]. Indeed, OVX, a model of menopause, increased body weight with significant fat accumulation (Figure 10). On the contrary, in males, the lack of testosterone after orchidectomy decreased the body weight without changes in fat content, most probably due to the lack of its anabolic effect on protein synthesis [326].

Besides the peripheral effect on metabolism (e.g., facilitation of insulin secretion, control of glucose availability, and shifting to the use of lipid as the main energy substrate), E2 also exerts central effects, largely through neural modulation, thereby regulating the energy management of the whole body [328].

Obesity, especially visceral fat, is a serious risk factor—among others—for cardiovascular events as well as for type 2 diabetes mellitus (T2DM) [329], also called cardiometabolic diseases [321]. Large scale randomized clinical trials have suggested that HRT may reduce insulin resistance and decrease the risk of developing T2DM [321]. Despite promising evidence, HRT use is not recommended in the primary prevention of T2DM due to the complex interplay between underlying conditions and other health risks (e.g., obese women with T2DM may have higher risk of developing venous thromboembolism). Finding novel SERMs with beneficial E2-like metabolic effects on the tissue of interest while antagonizing ERs in the breast and uterus is desired [330]. Indeed, in the OXV rat model, TMX as well as RLX reversed undesirable metabolic blood levels [331]. Moreover, a new SERM (GSK232802A) reduced body weight and adiposity in non-human OVX primates by suppressing food intake and increasing activity, suggesting its possible therapeutic usage in humans [332]. On the other hand, with phytoestrogens, there is a lot of uncertainty due to the relative abundance of different phytoestrogens in a given diet, the need for conversion to an active principle through the gut microbiome, the synergistic effect of different phytoestrogens, etc. [333]. Therefore, despite the positive association between, e.g., urinary excretion of isoflavones and lower T2DM risk in US women [334], there are no clear recommendations on their usage.

##### 6.2.2.3. Atrophy of the Outer Barrier (Skin, Mucosa) and Hair

Menopause is also associated with the deterioration in the condition of skin and hair. An increasing number of studies have found a link between a drastic decrease in E2 levels and damage in the cellular and homeostatic mechanisms of the skin [335]. Moreover, a decrease in E2 levels may also play a role in reduced skin elasticity due to loss of collagen and elastin fibers [336]. Additionally, E2 plays a pivotal role in the regulation of hyaluronic acid, mucopolysaccharides, and sebum production as well [337,338,339]. Due to these alterations, E2 deficiency may result in changes in the moisture content and hydration of the skin as well as of all urogenital tissue including the vulva, vagina, bladder, and urethra vaginal mucosa [340]. One of the most serious consequences is the urogenital atrophy (newly renamed as GSM, see Section 5.1), which may contribute to sexual dysfunction [183].

To prevent side-effects, local treatment with E2-containing creams is available; however, systemic administration of selective compounds can be also beneficial. For example, ospemifene, a SERM, during a 3-month treatment significantly improved GSM, sexual function, and quality of life in general [341]. Thus, it is recommended for treatment of dyspareunia, persistent or recurrent genital pain that occurs just before, during, or after sex [141]. Ospemifene had even less cardiovascular side-effects than other SERMs [342]; however, it might even worsen the hot flushes [223,224].

#### 6.2.3. Musculoskeletal System

As for the musculoskeletal system, both ERs are found in human skeletal muscle regardless of gender or age [343]. Thus, loss of ovarian estrogen can directly trigger changes in skeletal muscle. As a molecular mechanism, E2—through ERα and ERβ—contributes to the binding of myosin and actin [344]. E2 deficiency also has a major influence on growth hormone secretion, which contributes to a faster decrease in muscle mass as well [345]. These changes may also contribute to GSM during menopause [346]. However, one of the most characteristic effects of E2 deficiency is an increased risk of osteoporosis.

##### 6.2.3.1. Osteoporosis: Role of E2 in Bone Integrity

**Figure 11:**
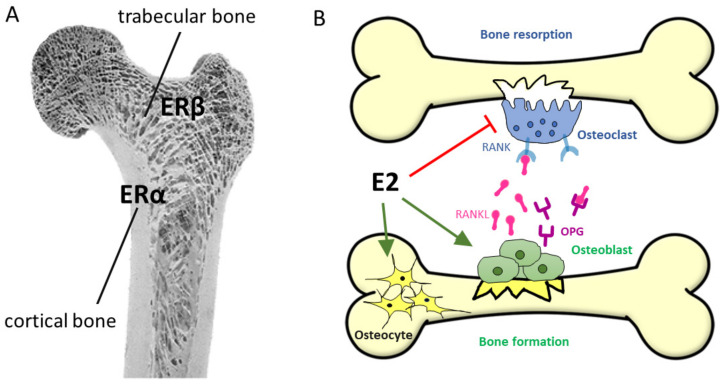
(**A**) Bone structure and the classical estrogen receptors on the bone. (**B**) Estrogen’s influence on bone formation. Green arrows indicate stimulation, while red bookend means inhibition of cell formation and survival. Abbreviations: E2—17β-estradiol; Erα—estrogen receptor alfa; Erβ—estrogen receptor beta; OPG—osteoprotegerin; RANK—receptor activator NF-κB; RANKL—RANK ligand.

Osteoporosis can be associated with several factors, such as menopause and aging, and is characterized by weakened bone microstructures with overall bone loss and excessive risk of fractures [347]. Every other postmenopausal woman has/will have an osteoporosis-related fracture [348]. In fact, the initial, first 3–5 years phase of postmenopausal osteoporosis (PMOP) might be linked to E2 deficiency [349] and is characterized by a rapid loss of trabecular bone structure, while the second stage, a slower period of 10–20 years, involves an age-dependent bone loss of both the cortical and trabecular bone structure, affecting women and men equally [350,351] (see Figure 11A).

HRT (either E2 alone or with progesterone) has been shown to prevent the bone loss and fracture not only in OVX mice [352], but also in women with PMOP [353]. The cellular basis of this effect is the fact that ERα and ERβ have been found in osteoblasts, osteoclasts, and osteocytes alike [351]. Bone formation and remodeling is regulated mainly by ERα, which mediates most of the effects of natural estrogen ligands, and is expressed primarily in cortical bone cells (Figure 11A). On the other hand, ERβ is expressed primarily in trabecular bone and may mediate the effects of phytoestrogens [37,354,355]. E2 increases osteoblast and osteocyte lifespans directly by inhibiting their apoptosis and regulating the osteoclast formation, survival, and activity [356,357] (Figure 11B). Moreover, indirect effects on osteoclast formation are mediated by the ERα-related effects of osteoblasts, as well as T-cells and B-cells [358]. E2 regulates the receptor activator NF-κB (RANK) ligand (RANKL), RANK, and osteoprotegerin (OPG) pathways. Indeed, these three molecules are essential, non-redundant factors for osteoclast biology [359]. Osteoclasts are entirely absent in RANK- or RANKL-deficient mice, leading to osteopetrosis, whereas OPG-deficient mice exhibit excessive bone resorption and severe osteoporosis. The main effect of E2 is the suppression of RANKL expression in osteoblasts, leading to reduced osteoclast activation [359]. The stimulated expression of OPG, a natural antagonist of RANKL, supports this process [360].

Although ERα, Erβ, and even the androgen receptor are present in cells of the bone formation, the antiapoptotic effects of sex steroids might be largely due to the MAPK signaling cascade with extranuclear activity [357] (Figure 5). This extranuclear mechanism of action can be modelled by estren, an ANGELS compound (see Section 5.4), which was shown to reverse E2-deficiency-induced bone loss through an antiapoptotic effect via the MAPK signaling pathway (Figure 5) [166]. However, this effect required the presence of the ligand binding domain of ERα and Erβ, suggesting that the non-genomic extranuclear effects of E2 are mediated by classical ERs, located near the plasma membrane [354]. However, the participation of GPER1 cannot be closed out through the activation of the MAPK, PI3K, and PKA pathways.

The best cure is the prevention, which—in the case of osteoporosis complication—includes the reducing of possible injuries (correcting impaired vision and/or hearing, minimizing fall-risk-inducing drugs (FRIDs), and establishing muscle and balance training), quitting smoking, and limiting alcohol intake [361]. Administrating adequate intakes of vitamin D, protein, and calcium as well as regular exercise with weight loss are recommended not only for prevention, but also for treatment [362,363]. However, in many cases, we cannot avoid specific medicine prescriptions. According to their mechanism of action, osteoporotic medications are divided into two categories: bone-resorption inhibitors and bone-formation supporters. Besides bisphosphonate—dominating the market at around 90% [364]—and anabolic agents, estrogen-like compounds are the best-known bone-resorption inhibitors [363]—approx. 5% of patients were administered it in South Korea [364]. During premature ovarian insufficiency, oral contraceptives are also in use [365]. However, due to the previously mentioned side-effects (see Section 4), more selective estrogen compounds such as SERMs are preferable [366]. TMX, as the first SERM, was extensively used, but nowadays RLX is preferable (see Section 5.1). However, long-term usage of bisphosphonates and SERMs in PMOP patients might result in serious adverse effects such as osteonecrosis of the jaw, atrial fibrillation, irregular vaginal bleeding, hot flushes, and atrophic vaginitis [347]. Moreover, bone-resorption inhibitors cannot reverse osteoporosis that has already progressed. Thus, further studies are needed to discover new treatment options. Basic and clinical research on estrogen and bone interaction not only expands our basic knowledge, but may also have a significant impact on the health of our aging population [367].

### 6.3. Role of E2 in Cancer

E2 may promote proliferation [368], and thus, increases the likelihood of developing cancerous processes in many organs, including the breast, ovaries, and endometrium, as well as the prostate [369]. Aberrant alternative splicing of ERs is also very common in cancer [370]. Even hepatocellular carcinoma shows strong gender dependence, with protective E2 actions [371]. In general, SERMs, acting on ERβ, might be beneficial.

#### 6.3.1. Breast Cancer

Breast cancer (BC) has three main types: the luminal BC, which is estrogen and/or progesterone receptor positive; the human epidermal growth factor 2 (HER-2)-positive BC; and the triple negative BC, which lacks the expression of these receptors [372]. Luminal BC is the most common type with the best prognosis [373], and it is usually treated with HRT [374]. In luminal BC, both ERα and ERβ are expressed. ERα acts as an oncogene and ERβ acts as a tumor suppressor [375]. Thus, the main goal of hormonal therapies is to suppress ERα activity in breast cancer cells. CYP19 (aromatase) inhibitors inhibit E2 production and lower the level of circulating E2, and thus, reduce ERα activity [376]. Some but not all estrogen-positive breast cancers express HSP27, an ERβ-associated protein; however, overexpression of HSP27 was found to be associated with both good and poor prognosis [377].

SERMs, such as TMX, bind to ERs and competitively inhibit E2 signaling [158]. SERDs, including fulvestrant, also bind to ERs and induce receptor degradation [378]. Due to the extremely positive clinical results, new SERMs and SERDs are currently being developed that may further increase the success of BC therapies [372].

#### 6.3.2. Ovarian Cancer

The importance of estrogen signaling in ovarian cancer (OC) is shown by the fact that ER-positive cases respond well to endocrine therapies, and that chronic E2 administration increases its risk [379]. Normal ovarian epithelial cells express mainly Erβ; however, its expression continuously decreases alongside OC progression [380].

On the contrary, significant ERα expression has been detected in OC, especially in endometrioid form, where the ratio of ERα-positive cases is over 80% [381,382]. E2 induces epithelial-to-mesenchymal transition and promotes cell migration through ERα [383]. Based on the foregoing, selective inhibition of ERα signaling may be a good therapeutic option for the treatment of ERα-positive OC. Indeed, several in vitro studies indicated that ERα-positive OC responds well to TMX and fulvestrant treatment, and their effects are mediated by ERα [384,385]. This is confirmed by clinical data showing that TMX and the CYP19 inhibitor letrozole were successful in the treatment of ERα-positive OC [379].

On the contrary, ERβ activity leads to decreased proliferation and apoptosis induction in OC [386]. Patients with ERβ-positive OC have better prognosis for survival [387]. Thus, selective activation of ERβ might be also beneficial. Indeed, a subtype-selective ERβ agonist [388] as well as two natural compounds with similar efficacy [389] significantly inhibited human ovarian cancer cell growth in vitro. Phytoestrogens (see Section 5.2) also have high affinity to ERβ [390]. Therefore, it is not surprising that genistein and daidzein as well as an ERβ agonist (ERB-041) significantly inhibited ovarian cancer cell migration, invasion, and proliferation, as well as inducing cell cycle arrest and apoptosis. These effects were due to the activation of alternative, non-classical pathways, such as PI3K (see Figure 5).

#### 6.3.3. Endometrial Cancer

E2 signaling is actively involved in the development of endometrial tumors (EC). Abnormally elevated E2 levels can cause endometrial hyperplasia, which can lead to endometrioid-type EC [391,392], which is the most prevalent one with the best prognostic outcome [391]. Traditionally, EC has been treated with surgery followed by radio- and chemotherapy [393]. However, for patients with metastases as well as those who want to maintain their fertility, hormonal therapies may be an alternative [394]. Most of the EC types express ERs (both ERα and ERβ) alongside progesterone receptors [395]. For hormonal therapies, progestin is the most commonly used, inhibiting the action of the ER through progesterone receptors [396]. More recently, SERMs, SERDs, and aromatase inhibitors have been increasingly studied to treat EC.

#### 6.3.4. Prostate Cancer

Controversially, female hormones (estrogens) have a significant role in male cancer development. In fact, the concept of “male” and “female” hormones is an oversimplification of a complex developmental and biological network of steroid actions that directly impacts many organs [397]. CYP19 (aromatase) is expressed in many organs and cells; thus, local production and action of E2 in men is likely physiologically relevant. It seems to be especially important for epiphyses fusion, normal bone density, as well as healthy metabolism. Moreover, even in males, E2 may provide significant feedback information at the pituitary level through ERα.

The first effective treatment against prostate cancer (PC) was androgen deprivation therapy performed by bilateral orchiectomy or E2 injection [398]. The antitumor effect of E2 therapy was achieved through reduced production of androgenic hormones via the negative feedback on the pituitary gland. The direct effects of E2 on prostate cells have been recognized only in recent years [399]. In the human prostate, ERα is expressed in stromal cells and in the androgen-independent basal cell layer [400]. Elevated ERα activity is considered to be carcinogenic. During malignant transformation of the human prostate cells, the expression of ERα increases and luminal cells also start to express the receptor [400]. ERα retains its increased activity until the late phase of human PC [401].

In the healthy human prostate, androgen-dependent luminal cells express ERβ [402]. In most PC types, the expression of ERβ is reduced or totally absent [403,404]. There is increasing evidence of the tumor-suppressor effect of ERβ activity inhibiting PC progression [405]. ERβ is also able to inhibit ERα signaling and induce apoptosis in PC cells [406]. However, ERβ may have some tumorigenic effect, supported by evidence about the overexpression of the receptor in metastatic PC cells in bone marrow and lymph nodes [402].

The role of the GPER1 in PC is still unclear. Expression of GPER1 has been shown in PC cells, and there is evidence of both its tumorigenic and tumor-suppressor effects. Clarifying its exact role requires further investigation [401].

Diethylstilbestrol (DES), a synthetic, non-steroidal estrogen, was commonly used as an estrogen therapy—among others—to treat castration-resistant PC (CRPC). Although DES has no beneficial effects in the early stage, it was beneficial in progressed metastatic CRPC [401]. Because the oral E2 therapies have highly toxic side effects, current clinical trials focused on transdermal administration of low-dose estrogens with positive outcomes [401,407,408]. Alternatively, SERMs and phytoestrogens may be also used in PC treatment. For example, toremifene, a second-generation SERM (see Section 5.1), reduced the five-year recurrence of bone metastatic PC [409]. According to in vitro results, RLX could be another promising SERM in PC therapies [410]. Natural phytoestrogens selectively bind to ERβ and induce anti-androgenic and protective effects in prostate epithelial cells [399,411]. It is also worth mentioning that in countries where the diet contains high phytoestrogen intake, the occurrence of prostate cancer is very low [412].

### 6.4. General Health Benefit: Antiviral Effect

Repurposing previously approved medications for new indications by taking advantage of off-target effects has gained traction, particularly in areas of medicine that do not offer large profits to pharmaceutical firms [413].

Before the COVID-19 pandemic, infectious disease discovery research had been declining among large pharmaceutical companies; therefore, at the time of the outbreak, the potential payoff of repurposing became attractive. As the SERM clomiphene was shown to have antiviral effects against the Ebola virus by interfering with virus entry into the target cell [414], the hypothesis arose that it might also be effective against COVID-19 infection [415].

## 7. Discussion

E2 is one of the most important female sex hormones and plays an important role in several tissues and organs not only in females, but also in males. E2 can affect reproduction and parturition through highly complex, non-classical signaling pathways in addition to the classical one, having both central (negative feedback on GnRH neurons and LH/FSH) and peripheral actions (direct effects in ovaries and uterus). The menopausal E2 depletion highlighted its role in thermoregulation, mood changes, memory deterioration, migraine, metabolic and cardiovascular changes, skin and hair aging, muscle degradation, and osteoporosis, and HRT was introduced. The side-effects of HRT showed the cancerogenic effect of E2 in the breast, ovarium, and endometrium, which called attention to the non-classical mechanism of action and alternative therapies. Mapping the non-classical effects helped to understand the role of E2 in terms of neuroprotection (anti-inflammatory and anti-apoptotic effects and the increasing of spike density), the cardiovascular system (vasodilatation), and osteoporosis (the increasing of bone mineral density, the inhibition of osteoclasts, and the modulation of osteoblasts, see Figure 11).

New, more selective modulators open the possibility of more effective interventions with less side-effects. Therefore, we might expect an increase in the indications of HRT even to men (e.g., for hypogonadism [180] or as a neuroprotective agent). However, we are still far from our ultimate goal.

## Figures and Tables

**Table 1:** Summary table about the tissue-specific action of E2 and other alternative estrogen-like compounds. The brain column includes the vasomotor symptoms (VMS). (+) Agonistic effect on ERs, (−) antagonistic effect on ERs, (−/+) controversial literature data, (?) not known.

Molecule	Brain (VMS)	Cardiovascular System	Bone	Breast	Endometrium	Therapy	References
Estrogen	+	+	+	+	+	Hormonal contraception, hormone replacement therapy (HRT), feminizing hormone therapy in transgender woman.	refer Section 6
Clomiphene citrate	−/+ (dose-dependent)	+	+	−	−	Treatment of female infertility, and male hypogonadism.	[111,112,113,114]
Tamoxifen	−	+	+	−	+	Adjuvant treatment of ER+ breast cancer, prevention in high-risked women.	[115,116,117,118,119,120,121,122,123,124,125,126,127,128,129,130]
Toremifene	−	+	+	−	+ (weak)	Adjuvant treatment of ER+ breast cancer.	[131,132,133]
Raloxifene	−	+	+	−	neutral	Treatment of osteoporosis in postmenopause, or in women with high risk of ER+ breast cancer.	[134,135,136]
Bazedoxifene	−	+	+	−	−	Treatment of osteoporosis in postmenopausal women.	[137,138,139,140]
Ospemifene	−	+	+	−	−/+	Treatment of genitourinary syndrome of menopause.	[141,142,143]
Lasofoxifene	− (weak)	+	+	−	−/+	Treatment of osteoporosis and vaginal atrophy.	[144,145]
Phyto-estrogens	+	+	+	−	−	Treatment of menopausal symptoms.	[146,147,148,149,150,151,152,153,154,155,156,157]
Fulvestrant	−	−	−	−	−	Treatment of advanced ER+ breast cancer.	[158,159,160,161,162,163,164]
ANGELS	+	+	+	?	−	Not in therapy.	[166,167,168,169,170]
G-1	+	+	+	+	+	Not in therapy.	[171,172,173,174,175,176]
G-15 and G-36	−	−	?	−	−	Not in therapy.	[176,177,178]

## Data Availability

Not applicable.
